# β-Adrenergic Inhibition of Contractility in L6 Skeletal Muscle Cells

**DOI:** 10.1371/journal.pone.0022304

**Published:** 2011-07-28

**Authors:** Anette I. Öberg, Nodi Dehvari, Tore Bengtsson

**Affiliations:** Department of Physiology, The Wenner-Gren Institute, Stockholm University, Stockholm, Sweden; Stockholm University, Sweden

## Abstract

The β-adrenoceptors (β-ARs) control many cellular processes. Here, we show that β-ARs inhibit calcium depletion-induced cell contractility and subsequent cell detachment of L6 skeletal muscle cells. The mechanism underlying the cell detachment inhibition was studied by using a quantitative cell detachment assay. We demonstrate that cell detachment induced by depletion of extracellular calcium is due to myosin- and ROCK-dependent contractility. The β-AR inhibition of L6 skeletal muscle cell detachment was shown to be mediated by the β_2_-AR and increased cAMP but was surprisingly not dependent on the classical downstream effectors PKA or Epac, nor was it dependent on PKG, PI3K or PKC. However, inhibition of potassium channels blocks the β_2_-AR mediated effects. Furthermore, activation of potassium channels fully mimicked the results of β_2_-AR activation. In conclusion, we present a novel finding that β_2_-AR signaling inhibits contractility and thus cell detachment in L6 skeletal muscle cells by a cAMP and potassium channel dependent mechanism.

## Introduction

Adrenoceptors (ARs) are G protein coupled receptors (GPCRs) expressed in virtually all organs in the body. They can be activated by the hormone epinephrine and the neurotransmitter norepinephrine and signal to several endpoints in different tissues. There are three pharmacologically distinct subtypes of ARs: α_1_-, α_2_- and β-ARs. The β-adrenoceptors (β-ARs) are currently classified into β_1_-, β_2_- and β_3_-ARs which are predominantly G_s_-protein-coupled receptors. The classical mechanism for signal transduction downstream of β-ARs is activation of adenylate cyclase (AC) which catalyzes the conversion of adenosine triphospate (ATP) to cyclic 3′,5′ adenosine monophosphate (cAMP). One of the major downstream effectors of cAMP is the serine/threonine protein kinase A (PKA). Upon increased levels in the cell, cAMP binds to the regulatory subunit of PKA which results in the dissociation and consequent activation of the catalytic subunit, which subsequently targets several proteins. It is also well documented that cAMP can act via the exchange factor directly activated by cAMP (Epac) [1], [2], a guanine nucleotide-exchange factor (GEF) that can activate Rap1, a small Ras-like GTPase involved in cellular functions such as cell proliferation, differentiation, apoptosis and adhesion. A third, less well-studied, target of cAMP includes ion channels that can be directly activated by cAMP binding. Nucleotide binding and activation of channels has been characterized in two protein families: the cyclic nucleotide-gated channels (CNG) and the hyperpolarization-activated cyclic nucleotide-gated (HCN) channels. Modulation of ion-transport by cAMP has also been proposed to occur with other Na^+^- and K^+^-channels [3], [4].

Addtionally, stimulation of β-ARs can also induce cAMP-independent signals. The most well described of these is the β-arrestin pathway, though βγ-dependent events have also been suggested.

Thus, the β-adrenoceptor is capable of activating multiple signaling cascades, leading to several cellular and physiological endpoints. In skeletal muscle systems, several β-adrenergic effects have been described: increased protein synthesis and reduced protein degradation, both of which lead to increased muscle mass; potentiation of muscle twitch; enhanced activity of ion channels, increased lipolysis via hormone sensitive lipase (HSL); enhanced glycogen metabolism, and; increased glucose uptake. The latter endpoint has been the subject of several studies and seems to be regulated by the β_2_-AR via both cAMP-dependent and independent pathways. In the L6 cell line, a commonly used model system for skeletal muscles, β-adrenergic stimulation induces glucose uptake though multiple signaling pathways [5], in which, glucose uptake is only partly blocked by cAMP-inhibition [5], suggesting the involvement of atypical signaling.

In the current paper we present evidence that the β-adrenergic pathway affects cell morphology and contractility. In the following text we have defined contractility as the ability of cells to contract/becoming shorter, both regarding muscle and non-muscle cells. We primarly investigated the effect of β-AR signaling on contractility in skeletal muscle, using the L6 cell line. For this, we employed an assay in which the cells were treated with the calcium chelator EDTA or calcium-free PBS. Removal of extracellular Ca^2+^ has been used in several studies as a model system for cellular contraction [6]–[10]. Though the mechanism is not fully elucidated, it is shown to be myosin II dependent [11] and has been reported to be caused by increased membrane Na^+^ permeability [12]. It has also been shown that channels that normally transport Ca^2+^ can, in the absent of Ca^2+^ ions, transport Na^+^ across the plasma membrane [13]. In Caco-2 intestinal epithelial cells, openings in tight-junctions upon treatment with calcium-free buffer was proposed to be dependent on myosin light chain kinase, MLCK [7]. However, in several other studies, these breaches were instead shown to be caused by the Rho A-ROCK signaling pathway: in T84 and SK-CO15 human colonic epithelial cells, contraction was dependent on the Rho A – ROCK pathway activated by GEF-Hi [10] and, in rat brain endothelial cells, depletion of extracellular Ca^2+^ induced cell rounding in a RhoA-dependent manner [14]. In the present study, we show ROCK to be important for calcium depletion-induced contractility in L6 myotubes. By using this calcium-depletion model, we show that stimulation of β-AR can inhibit cell contractility and detachment. We also show that this signal is cAMP dependent but independent of the classical downstream effectors PKA and Epac. Furthermore, involvement of PKG, PI3K and PKC was ruled out. We show for the first time that the mechanism is mediated by potassium channels.

## Results

### Stimulation of β_2_-adrenergic receptors inhibits calcium depletion-induced cell detachment

**Figure 1:**
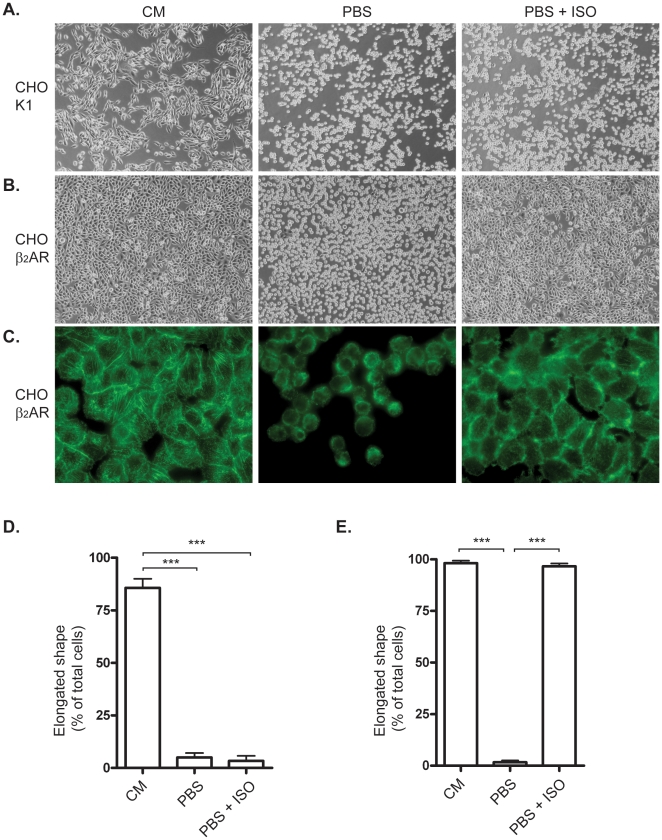
β_2_-adrenoceptor-mediated inhibition of cell detachment in CHO cells. A) CHO-K1 cells were kept in culture media (CM) or phosphate buffered saline (PBS) for 20 min with or without 30 min of pre-treatment with 1 µM isoprenaline (ISO). B) CHO cells stably transfected with human β_2_-adrenoceptor in CM, PBS or PBS+ISO. C) FITC-Phalloidin-stained CHO β_2_-AR cells in CM, PBS or PBS+ISO. D) Morphology of CHO-K1 cells described by counting cells with elongated shape. The bars represents percentage of total cells. E) Morphology Quantification of CHO-β_2_ cells. Histograms show percentage of total cells expressed as mean ±SEM of three independent experiments. *** p<0.001, one-way ANOVA.

To verify that removal of extracellular Ca^2+^ would lead to changes in cellular shape as has been previously described [6], [12] we first employed CHO-K1 cells as a model system [15]. CHO cells were subjected to calcium-free Phosphate buffered saline (PBS) and after 20 minutes, all cells displayed sperical morphology (Fig. 1A), confirming that CHO-K1 cells can be used in this assay. CHO-K1 cells express very few or no β_2_-ARs under normal conditions, and treatment with the β-AR agonist isoprenaline had no effect on these cells (Fig. 1A). These observations were also quantified by counting elongated versus spherical cells, and the results in figure 1D show that following exposure to PBS, nearly 100% of the cells displayed a spherical morphology.

To investigate if β_2_-ARs could be involved in governing cellular shape we used CHO cells stably transfected with human β_2_-AR and tested these cells in our assay. After 20 minutes exposure to calcium-free PBS, nearly all CHO β_2_-AR cells displayed spherical morphology (Fig. 1B). We were surprised to find that with β-AR agonist (isoprenaline) present in the calcium-free PBS the spherical morphology was almost fully prevented and the cells displayed the same (elongated) shape as control cells (Fig. 1E). These observations were quantified by counting cells with spherical versus elongated shape. Figure 1E shows that after exposure to calcium-free PBS, the cells have close to 100% spherical shape but when treated with isoprenaline, almost 100% of the cells kept their elongated shape, despite the exposure to PBS. These results show that the isoprenaline can prevent the rounding of the cells and that the effect is meditated by the β_2_-AR. Confirming experiments were done in CHO-cells transiently transfected with human β_2_-AR, and here, too, isoprenaline prevented the rounding of the calcium-free PBS treated cells (data not shown). We also noted that upon prolonged exposure to calcium-free PBS (about 1 h) the cells rounded and finally detached from the cell plate and that this could be prevented with isoprenaline treatment. We also noted that isoprenaline-treated cells eventually detached, but the effect of isoprenaline lasted as long as 24 h (data not shown). This indicated that activation of β_2_-AR could prevent both the spherical shape and detachment. To better visualize the effect, the CHO β_2_-AR cells were stained with FITC-phalloidin. Figure 1C confirms that PBS treatment results in spherical morphology as well as detachment of the cells, which is also indicated by the loss of stress fibers. These changes were effectively prevented by isoprenaline treatment.

**Figure 2:**
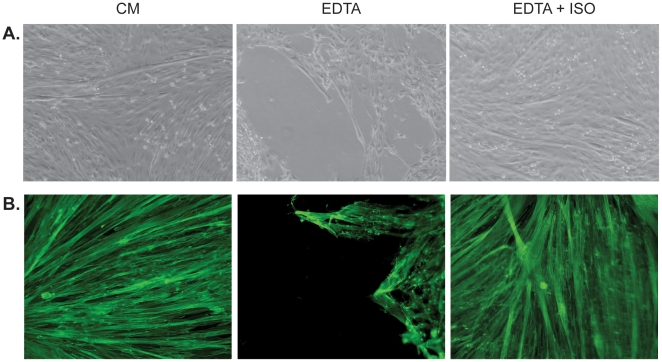
Cell detachment induced by EDTA was inhibited in L6 cells by isoprenaline. A) Differentiated L6 myotubes were kept in culture media (CM) or EDTA with or without 1 µM isoprenaline (ISO) for 20 min. The isoprenaline stimulated cells were pre-treated with this drug for 30 min before EDTA exposure. B) FITC-Phalloidin-stained L6 myotubes in CM, EDTA or EDTA+ISO. Representative pictures from 3–6 independent experiments.

Since CHO cells express negligible amount of β_2_-AR we wanted to test a model system with endogenous β_2_-AR expression. L6 skeletal muscle cells express endogenous functional β_2_-ARs and have been used for studies of β-adrenergic effects on muscle [5], [16]–[18]. In skeletal muscle, β-ARs are considered to be the most important of the adrenoceptors with β_2_-AR as the predominant subtype, and this is also true for the L6 rat skeletal muscle cells [16], [17]. Upon switching to low serum, L6 rat skeletal muscle cells differentiate so that the myoblasts fuse to become large multinucleated myotubes (Fig. 2A).

When exposed to calcium-free PBS or calcium chelators BABTA, ethylene glycol tetraacetic acid (EGTA, not shown) or EDTA (Fig. 2A and Fig. S1) in the media, the cells contracted and detached from the surface of the dish. Calcium depletion has previously been used in endothelial cells but we show for the first time that this method can also be utilized in skeletal muscle cells. Myotubes consist of a large number of fused myoblasts, which are highly elongated in shape; under the described conditions, and contraction of the myotubes leads to detachment from the growth surface. After 10 min exposure to PBS, EDTA or BAPTA, the L6 myotubes contracted so that only 50% of the bottom of the well was covered with cells and after 20 min this number had decreased to around 25%. However, for cell that were pre-treated with isoprenaline, and kept in PBS, EDTA or BAPTA with continuous presence of this drug, the corresponding numbers was 100% after 10 min and 95% after 20 min.

Addition of β-AR agonist isoprenaline to all treatments inhibited detachment of both myoblasts and myotubes (not shown, Fig. 2 and Fig. S2). We have furthermore confirmed the same effect in human primary skeletal muscle cells and C2C12 mouse skeletal muscle cells (data not shown). Filamentous actin staining with FITC-conjugated phalloidin was also performed in L6 myotubes and results show that PBS treatment leads to cell detachment and isoprenaline treatment prevents this effect (Fig. 2B).

### Calcium depletion induces contractility leading to cell detachment in L6 myotubes

**Figure 3:**
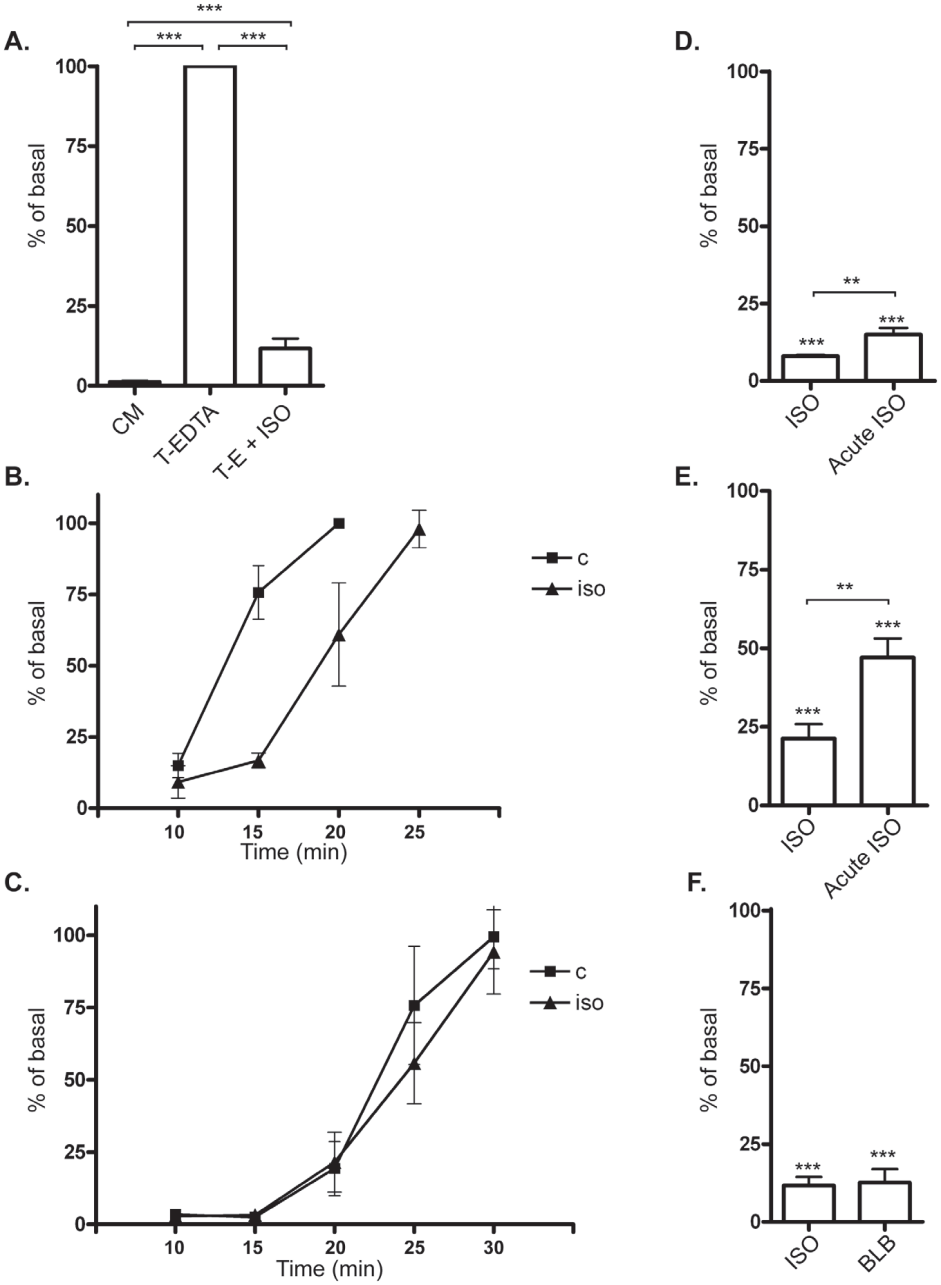
A quantitative cell detachment assay was used to investigate the involvement of myosin contractility in differentiated L6 cells. The amount of detached cells is expressed as % of basal where basal means cells that are not treated with any drugs before exposure to trypsin·EDTA for 20 min. A) Cells were either kept in culture media (CM) during the whole experiment or expored to Trypsin·EDTA (T-E) for 20 min. T-E+ISO indicated cells pre-treated for 30 min of 1 µM isoprenaline (ISO) before the 20 min exposure to T-E in the continuous presence of isoprenaline (***). Histograms show percentage of unstimulated cells detached by T-EDTA expressed as mean ±SEM of four independent experiments. ** p<0.01, *** p<0.001, one-way ANOVA versus control cells in T-EDTA. B) L6 myotubes kept in Trypsin·EDTA with (triangels) or without (squares) isoprenline treatment. Aliquots of the detached cells were harvested and counted at the timepoints 10, 15, 20 and 25 min. C) L6 myoutbes kept in trypsin alone with (triangles) or without (squares) isoprenaline treatment. Aliquots of the detached cells were harvested and counted at the timepoints 10, 15, 20, 25 and 30 min. D) L6 myotubes were kept in Trypsin·EDTA for 20 min after 0 or 30 min pretreatment with isoprenaline. In both C and D, point show % of cells detached after 20 min in Trypsin·EDTA. *** p<0.001 with one-way ANOVA for iso and acute iso versus control. ** p<0.01 for iso versus acute iso. E) L6 myoblasts were kept in Trypsin·EDTA for 3 min after 0 or 30 min pretreatment with isoprenaline. *** p<0.001 with one-way ANOVA for iso and acute iso versus control. ** p<0.01 for iso versus acute iso. F) 30 min pre-treatment of 50 µM of the myosin-inhibitor blebbistatin (blb) or 30 min of 1 µM ISO pre-treatment followed by 20 min of T-E exposure. *** p<0.001, one-way ANOVA versus T-EDTA.

Since addition of EDTA detached the cells from substrate but did not separate L6 myotubes from each other, a combination of EDTA and trypsin was used in order to obtain single cells. This method made it possible to count the cells and thus quantify the detachment. Upon exposure of L6 myotubes to trypsin·EDTA for 20 min, about 100% of cells had detached (Fig. 3B). Thus in the following experiments, supernatant with detached cells were collected after 20 min in trypsin·EDTA. Addition of isoprenaline reduced detachment to about 10% of the control cells (Fig. 3A and 3B). When L6 mytoubes were exposed to trypsin alone, they detached slower than by the combination of trypsin and EDTA (Fig. 3C), and in contrast to the EDTA-effect, detachment caused by trypsin was not inhibited by isoprenaline treatment. These results imply that the β-adrenergic effect is independent of adhesion processes involving, for example, integrins.

Isoprenaline treatment inhibited trypsin·EDTA induced contraction in both myoblast and myotubes, while the effect was much more pronounced in the latter system (Fig. 3D and 3E). Pretreatmant with isoprenaline for 30 min enhanced the effect, but also acute addition of the drug togheter with trypsin·EDTA gave a significant effect.

In order to study the properties of the detachment of the L6 cells, we investigated the role of myosin, which is highly expressed in these cells. It is well known that contraction can result in cell detachment [6]. Contractility in cells is dependent on the motor-protein myosin which operates via interaction with actin in the cytoskeleton.

The non-muscle myosin II (NMII) blocker blebbistatin was used in order to inhibit contractility without causing disassembly of the actin cytoskeleton. Results show that blebbistatin inhibited the EDTA/calcium depletion-induced cell detachment, confirming the dependence of the contractile machinery (Fig. 3F).

**Figure 4:**
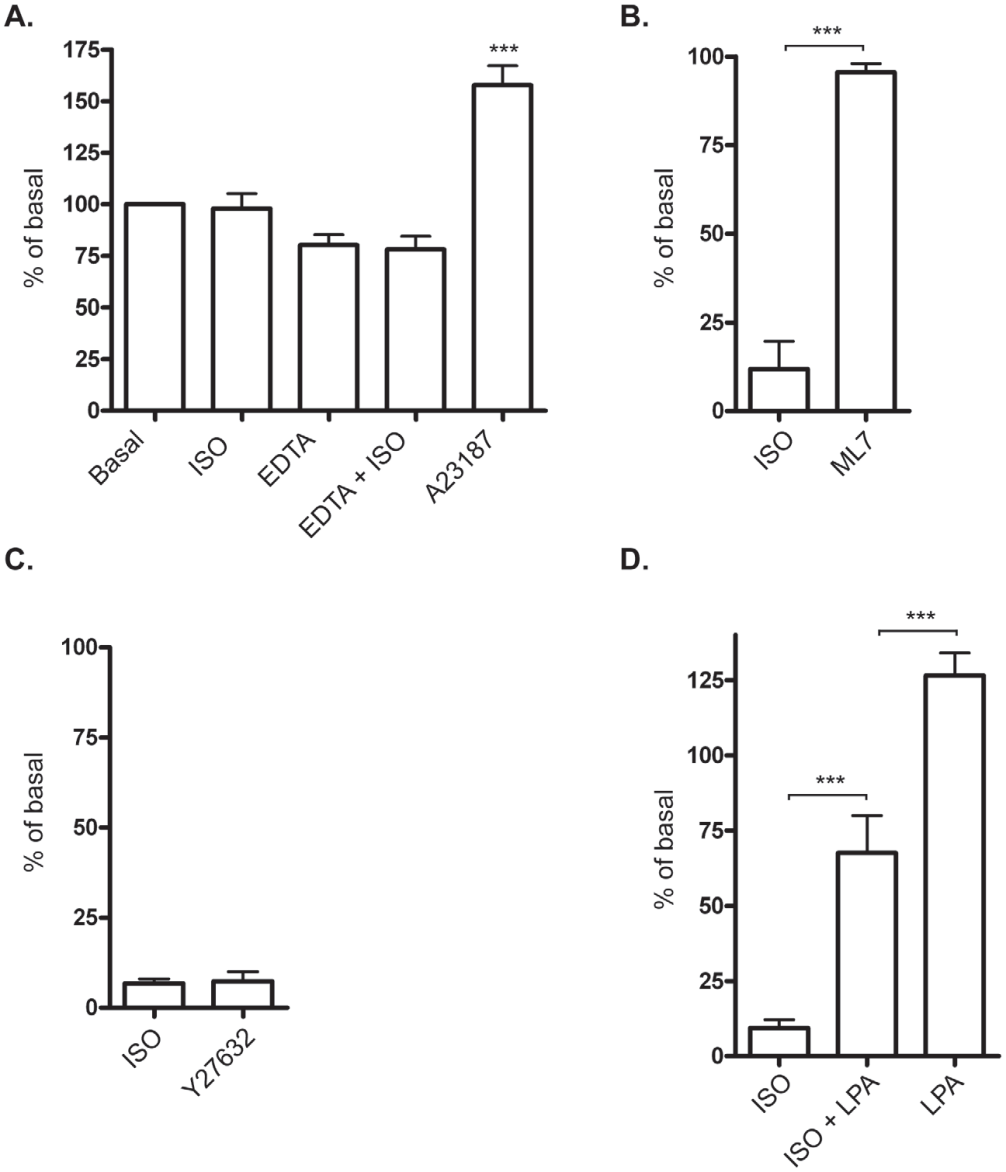
Characterization of the cell-detachment mechanism in L6 mytoubes. A) Intracellular levels of calcium were detected in L6 myotubes by the calcium-sensitive fluorescent dye Fluo-4. Control cells and cells pre-treated with 1 µM isoprenaline (ISO) were exposed to 1.6 mM EDTA for 2–6 min. 10 µM of the ionophore A23187 (A23) was used as a positive control. N = 4, *** p<0.001 one-way ANOVA for A23187 versus basal. B) Detachment assay with cells pre-treated for 30 min with 1 µM isoprenaline (ISO) or 15 µM ML-7. Histograms show percentage of control cells detached by T-EDTA expressed as mean ±SEM. N = 3. *** p<0.001, one-way ANOVA versus ISO. C) 20 µM Y-27632 and D) 20 µg/ml LPA followed by 20 min exposure of Trypsin·EDTA. N = 3–4, *** p<0.001 one-way ANOVA versus ISO or ISO+LPA.

To monitor the potential effect of the EDTA-treatment on intracellular calcium levels, which could potentially affect contractility, L6 myotubes were loaded with the green-fluorescent calcium indicator Fluo-4. After 2 min in EDTA, when cells started to contract, there was no significant change in intracellular calcium levels (Fig. 4A). Isoprenaline treatment did not affect the calcium levels, neither in cells kept in media nor during EDTA exposure. The ionophore A23187, that is known to induce a rapid increase in intracellular calcium, was used as positive control. This treatment increased calcium levels significantly, verifying the reliability of the system.

The contractility of NMII is regulated by phosphorylation of the regulatory light chain, and the major kinase involved is myosin light chain kinase (MLCK) a Ca^2+^-calmodulin-dependent enzyme. Since MLCK has been reported to be activated upon calcium depletion [7], we investigated the involvement of this kinase with a pharmacological blocker, ML-7. Cells detached in the presence of the ML-7 (Fig. 4B) indicating that the regulation of NMII is not dependent in this study.

There are also other kinases such as Rho-associated kinase (ROCK) and citron kinase that can phosphorylate NMII. ROCK, in particular, can also regulate NMII indirectly via inhibition of MYPT [19], and depletion of extracellular Ca^2+^ has been shown to induce Rho A and ROCK activation in both colonic epithelial and brain endothelial cells [10], [14]. To test if ROCK is necessary for contractility in our system we used the inhibitor Y-27632 to block ROCK activity. Figure 4C shows that detachment of cells induced by EDTA was blunted in response to Y-27632, confirming the importance of ROCK for contractility. Another approach is to study activation of Rho-ROCK by using Lysophosphatidic acid (LPA), a small, ubiquitous glycerophospholipid that has been used by other investigators to show the involvement of ROCK-mediated contractility. LPA has five receptor subtypes: LPA1–LPA5, which are encoded by genes named *LPAR1*–*LPAR5* in humans and *Lpar1*–*Lpar5* in mice. All five receptors are type I, rhodopsin-like GPCRs but differ in their tissue distribution and downstream signaling pathways [20]. Most mammalian cells exhibit varied responses to LPA (e.g. proliferation, cell migration or smooth muscle contraction) depending on cell type [20]. LPA activates RhoA and is known to induce cell morphology changes, increase endothelial permeability and inhibit gap-junctional communication between adjacent cells [21]. Our results showed that LPA inhibited the isoprenaline effect, resulting in cell detachment despite the adrenergic stimulation (Fig. 4D). This indicates that adrenergic signaling interacts with the Rho-ROCK pathway. There was no significant difference between LPA-treated and control cells after 20 min in trypsin·EDTA, however we noted that at earlier time points that LPA-treatment enhanced the speed of contraction. Taken together, our results support previous findings that calcium depletion induces contractility via ROCK pathway.

### β-adrenergic inhibition of contractility in a dose-dependent manner

**Figure 5:**
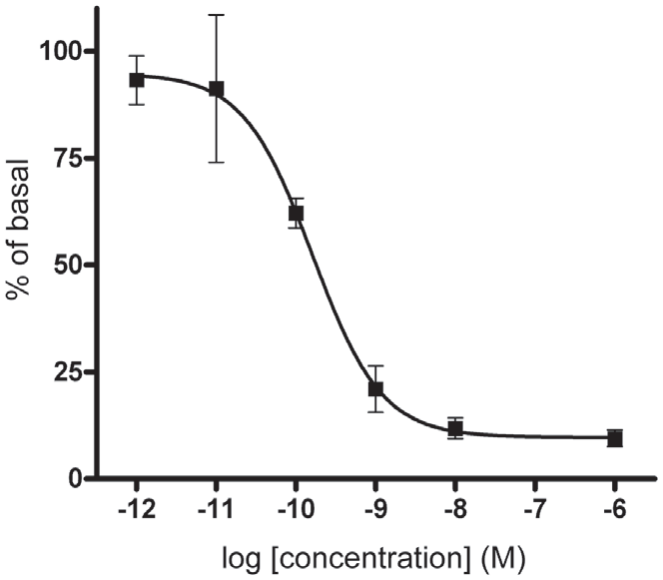
Isoprenaline inhibits contractility in a dose-dependent manner. L6 myotubes pre-treated with different concentrations of isoprenaline for 30 min followed by 20 min exposure to Trypsin·EDTA. Dose response curve show percentage of T-EDTA expressed as mean ±SEM of six independent experiments.

By using different concentrations of isoprenaline we show that β-AR inhibition of contractility follows a Michaelis-Menten-shaped dose-response curve (Fig. 5). Very low doses of isoprenaline had an effect, indicating this to be a very sensitive system with a pEC_50_–value at 9.7±0.4. The effect of β-adrenergic signaling can have different sensitivity depending on which endpoint is studied. Our previous results in L6 myotubes show that isoprenaline-stimulated glucose uptake has a pEC_50_ at 8.99 [17], whereas the endpoint of glycogen synthesis was less sensitive with a pEC_50_ at 7.46 [18].

### β_2_-adrenergic receptor-mediated regulation of contractility in L6 rat skeletal muscle cells is not dependent on the classical β_2_-adrenergic signaling pathway

**Figure 6:**
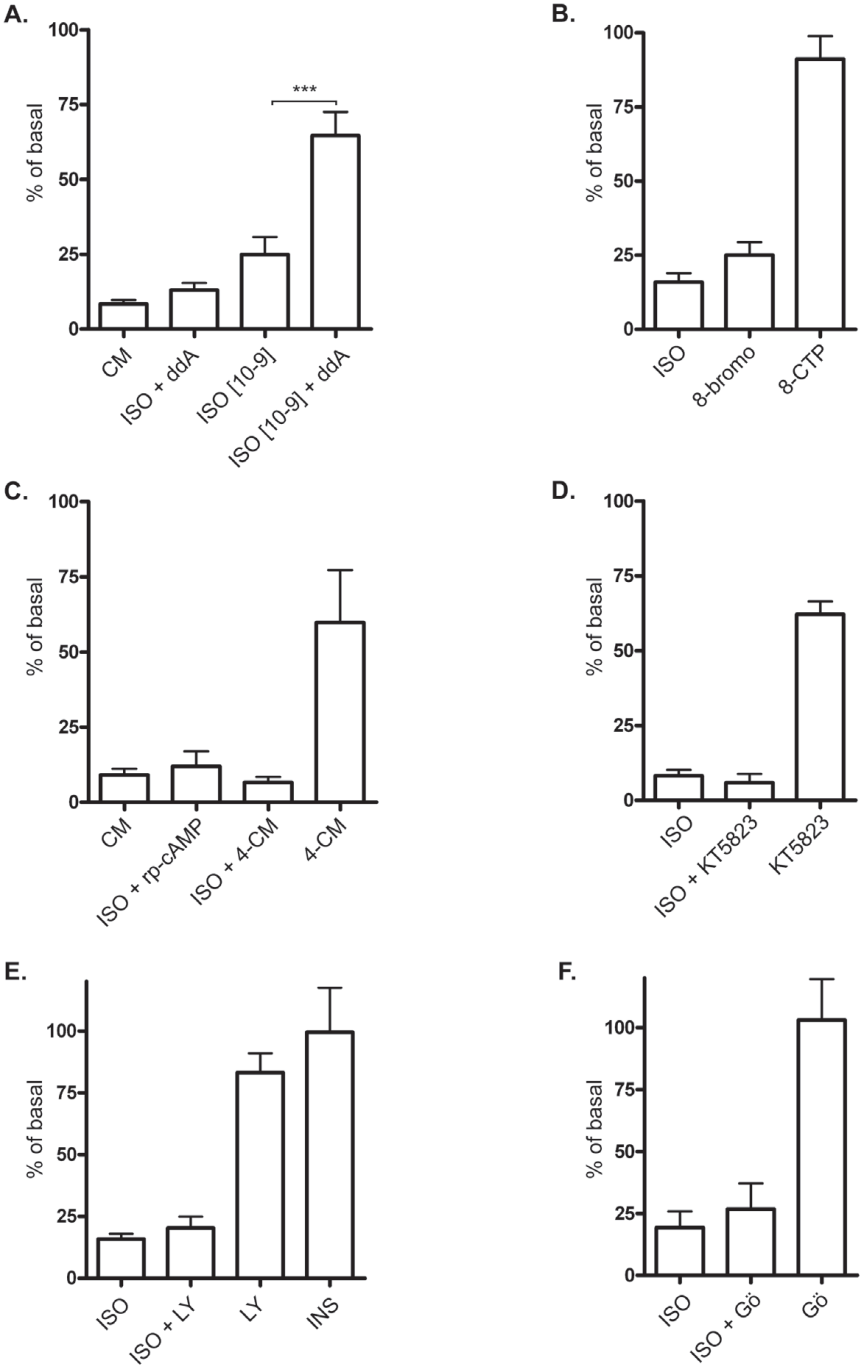
Isoprenaline inhibits contractility via cAMP in differentiated L6 cells. A) L6 myotubes were pre-treated with 50 µM ddA for 30 min before stimulation with 1 µM or 1 nM isoprenaline (ISO), 30 min following 20 min Trypsin·EDTA exposure. Histograms show percentage of control cells detached by T-EDTA expressed as mean ±SEM of three independent experiments. *** p<0.001, one-way ANOVA versus ISO. B) L6 cells were stimulated with 1 µM ISO or 1 mM of the cAMP-analogues 8-bromoadenosine-3′,5′-cyclic monophosphate, 8-bromo (30 min), N^6^-benzoyladenosine-3′,5′-cyclic monophosphate, or 5′- cyclic monophosphate, 8-CPT (30 min) followed by treatment of Trypsin·EDTA. N = 3. C) 1 µM ISO, 20 µM rp-cAMP (30 min before addition of iso) and 10 µM 4-CM (30 min before addition of iso) N = 3 D) 1 µM ISO and 10 µM KT 5823 (30 min before addition of iso) E) 1 µM ISO, pre-treatment with the LY294002 (LY, 30 min before addition of iso) or 1 µM insulin (INS, 30 min) N = 2–3 F) 1 µM ISO, pre-treatment with 5 µM Gö9683 for 5 min before addition of isoprenaline followed by 20 min exposure of Trypsin·EDTA, N = 2–3.

To investigate the mechanism of β-adrenergic inhibition of cell contractility and detachment we studied downstream signaling of β-AR. As described above, activation of β-ARs leads to G_s_-mediated stimulation of adenylate cyclase (AC), elevated cAMP levels and activation of PKA. The partial AC inhibitor ddA showed a tendency to mitigate the cell contractility/detachment inhibition caused by 1 µM isoprenaline. However, when used with 1 nM isoprenaline (a lower, but effective dose), ddA significantly counteracted the isoprenaline-induced effects (Fig. 6A). This shows that AC is involved and that probably only a small amount of cAMP is enough to inhibit EDTA induced contractility.

To study the direct effects of cAMP, we used the cAMP analogue 8-bromo-cAMP. Figure 6B shows that 8-bromo mimicked the effect of isoprenaline almost completely, further indicating cAMP dependence.

The traditional cAMP effector is PKA, but also Epac has also been shown to be a dominant effector. Epac-activator 8-(4-chlorphenylthio)-2′-O-methyladenosine-3′,5′-cyclic monophosphate (8-CPT) was used but did not have any significant effect on cell contractility and detachment (fig. 6 B) ruling out Epac as a candidate.

PKA inhibitors 4-CM and rp-cAMP were used, but neither could inhibit the isoprenaline effect (Fig. 6C), even at lower isoprenaline concentrations (data not shown). Instead, 4-CM mimicked the isoprenaline effect.

Another alternative signal would be activation of PKG, since this kinase has been shown to act downstream of cAMP to control contraction of smooth muscle cells [22]. We used the blocker KT 5823 to test PKG involvement, and subsequently ruled out PKG as the isoprenaline effect was unaltered.KT5823 had in itself an inhibitory effect on cell detachment.

Our previous studies of β-AR signaling in L6 myotubes show that PI3K, or related kinases, act downstream of β_2_-AR, leading to endpoints including glucose uptake and glycogen synthesis [5], [17], [18]. PI3K has been implicated in several pathways controlling key function of the cell, including motility, adhesion and actin remodelling, and has also been shown to be important for Ca^2+^-dependent contraction in smooth muscle cells [23]. We used the PI3K inhibitor LY294002; however, the results do not show any involvement of PI3K (Fig. 6E). Consistent with these findings, treatment with insulin could not inhibit calcium depletion-induced contractility in L6 cells (Fig. 6E).

Another kinase involved in the regulation of cell adhesion and contractility is PKC. In some systems, PKC enhances myosin contractility via the PKC-potentiated myosin phosphatase inhibitor, CPI-17. In smooth muscles with high CPI expression, PKC-activating agents can induce contraction; in species missing CPI, the PKC activation instead reduces contraction [24]. Additionally, in many non-muscle cells, PKC inhibits contraction via phosphorylation of myosin at ser1, ser2 or thr9 [19]. This is likely to be the case for skeletal muscle models such as L6 since CPI-17 is only expressed in smooth muscles cells [25]. To test this hypothesis, L6 myotubes were treated with PKC inhibitor Gö6983 but results (Fig. 6 F) show that inhibition of PKC could not inhibit the isoprenaline effect, suggesting that PKC is inessential in the present pathway.

### The β_2_-adrenergic receptor-mediated inhibition of contractility is dependent on potassium-channels

**Figure 7:**
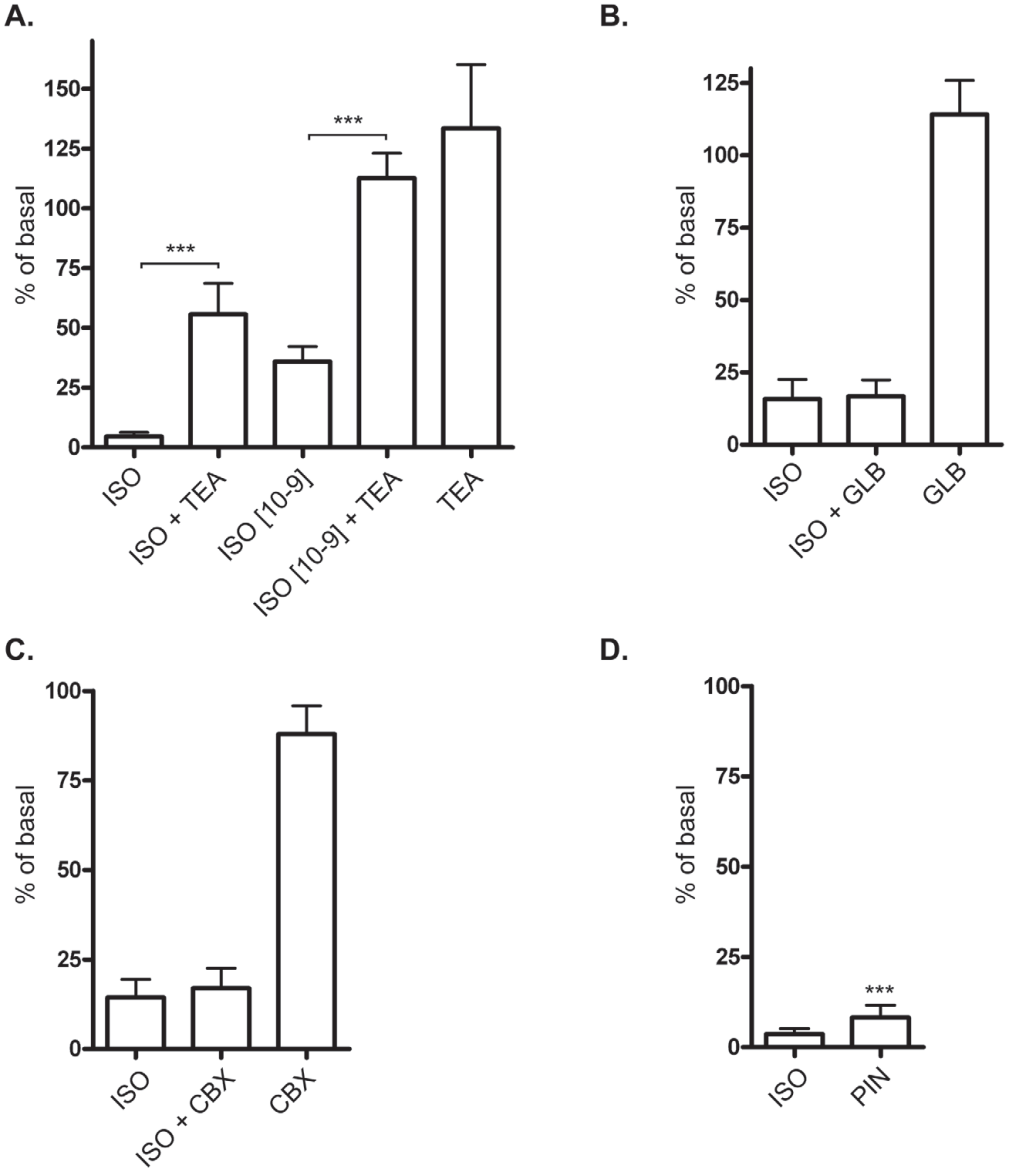
Potassium channels inhibit calcium depletion-induced contractility. A) L6 myotubes were pre-treated with 100 mM tetraethylammonium (TEA) for 10 min prior to 1 µM isoprenaline (ISO). Histograms show percentage of T-EDTA expressed as mean ±SEM. N = 3–4. *** p<0.001, student t-test versus ISO. B) 10 min exposure of 100 µM glibenclamide (GLB), N = 3 C) 10^−7^ M charybdotoxin (CBX) treated for 10 min N = 3 D) 100 µM pinacidil (PIN) for 10 min followed by Trypsin·EDTA exposure for 20 min. N = 3–4, *** p<0.001 versus Trypsin·EDTA.

β-adrenergic stimulation has been shown to affect potassium-channels in some systems [4], [26]–[28]and to investigate the possible role of potassium channels in the current signal, we used the general K^+^-channel blocker, Tetraethylammonium (TEA). We found that TEA could mitigate the 1 µM isoprenaline effect and completely prevent the 1 nM isoprenaline effect (Fig. 7A).

We tested glibenclamide, an ATP-sensitive potassium channel inhibitor, and charybdotoxin (CBX), an inhibitor of calcium-activated potassium channels as well as some classes of voltage-gated potassium channels. It has been previously shown that glibenclamide can inhibit the β-adrenergic effect in smooth muscle [26], and also in mesenteric arteries [28]). Yet, in rat pulmonary arteries it did not affect isoprenaline-mediated relaxation [27], and the same article reported that CBX had a partial effect on the β-adrenergic pathway [27]. In our experiments neither glibenclamide nor CBX could mimic or inhibit the isoprenaline effect, thereby ruling out these classes of potassium channels as potential candidates (Fig. 7B, C). Further investigations were done with the potassium-channel agonist pinicidil, which has previously been used to show that stimulation of the β-adrenergic pathway induced K^+^ channel activity in rabbit coronary smooth muscle cells [4]. Pinacidil inhibited contractility and detachment in response to calcium depletion, to nearly the same extent as isoprenaline (Fig. 7D), which corroborates our previous findings indicating that potassium-channels are involved.

## Discussion

β-adrenergic signaling exerts numerous effects in various tissues. In the present study we have investigated the effect of β-adrenergic signaling on cell detachment and contractility. Starting out with CHO cells, which express negligible quantities of endogenous adrenoreceptors, we utilized a calcium-depletion model to induce cellular contractility. When treated with calcium-free PBS, CHO cells round up to finally detach. In CHO cells with native receptor expression, treatment with the β-AR agonist isoprenaline had no effect on the morphological change seen after depletion of extracellular calcium; in CHO cells transfected (either stably or transiently) with the β_2_-AR, isoprenaline treatment prevented cell rounding. Thus, isoprenaline treatment has a clear effect on cell morphology and this effect is dependent on the β-AR. CHO cells are easily transfectable and were therefore suitable for this experiment.

For further experiments, however, we used a more physiologically appropriate model, the L6 skeletal muscle cells, which express β_2_-AR endogenously. We have an interest in the β-AR pathway. Specifically, the atypical signaling of β-AR to different endpoints has previously been investigated by us and others by using L6 skeletal muscle cells [17], [18], [29]. The present study has never been performed in a skeletal muscle model system.

We developed a cell detachment assay to quantify the number of cells that detach from the dish by using a combination of trypsin and EDTA. The detachment of cells from the dish can depend either on 1) direct inhibition of adhesion or 2) induced contractility, and subsequent loss of adhesion.

Adhesion is mediated through interaction of integrins with extracellular proteins. Trypsin treatment of cells effectively removes these connections but we demonstrated that they are unlikely to be involved in the β-adrenergic effect on cell contractility and adhesion, because isoprenaline had no effect on detachment induced by trypsin alone. Furthermore, the isoprenaline effect was more pronounced in differentiated L6 myotubes than in myoblasts. Myotubes contain more myosin and this goes in line with our hypothesis that myosin is important in the current process.

Although it is possible that isoprenaline could affect both integrins and contractility, our results strongly support the idea that the latter mechanism is the most important in our system.

The cell's contractile machinery is driven by a dynamic interaction between actin filament and myosin. There are about 20 classes of myosin of which class II is the largest and most well known. To this class belong the different myosin isoforms responsible for contraction of cardiac, skeletal and smooth muscles, but also the non-muscle myosin, NMII, which is found in all eukaryotic cells. Thus, muscle cells express both muscle specific and non-muscle isoforms of myosin. Non-muscle myosin is important for cell-cell and cell-matrix adhesion, migration and cytokinesis [19]. Mechanical properties of a single cell such as contractile forces are important for cell migration, neurite extension, cytokinesis, muscle cell contraction, cell cycle progression, angiogenesis, and differentiation [30]–[36].

In the present study we establish that myosin-based contractility is involved in contraction and detachment upon calcium depletion since inhibition of myosin with blebbistatin yielded results similar to those obtained with isoprenaline. L6 skeletal cells express a high level of myosin, which makes our model system suitable for studying contractility. We further investigated the contractile machinery activated by calcium-depletion by ruing out the possible role of MLCK, a kinase which regulates myosin by phosphorylation. We proceeded to investigate another kinase, ROCK, which also regulates myosin both directly, by phosphorylation, and indirectly, by regulating a phosphatase, MLCP, that in turn dephosphorylates myosin. Our results show that in our system, calcium-depletion induced cell detachment via a ROCK-dependent mechanism. Taken together our results suggest that it is more likely that isoprenaline acts on the contractile machinery than on the integrin system.

To study the pharmacological features of the isoprenaline effect on contractility and cell detachment we performed a dose-response study. These results revealed a highly sensitive system in which low concentrations of the β-adrenergic agonist isoprenaline potently effected contractility. In CHO cells, the calcium-depletion response was weaker than in L6 cells, which could be explained by the higher expression of myosin in L6. Additionally, several L6 cells fuse to become large myotubes which may accentuate and accelerate the effect, due to leverage, and could be the reason for the high sensitivity shown in the dose response curve.

We also investigated the classical downstream effectors of β-AR in order to further understand the intracellular signal leading to inhibition of contractility. By using the partial inhibitor of the cAMP-producing enzyme adenylate cyclase (ddA) as well as a cell permeable cAMP analogue (8-bromo-cAMP) we demonstrated that cAMP is indeed involved in this system. Although, there are several signaling cascades downstream of β-ARs, cAMP formation is still considered to be one of the major events.

Several reports show that cAMP can affect cell morphology in different model systems [37]–[39]. Increased cAMP-levels are also shown to promote cell adhesion via Epac and Rap1, in Ovcar 3 cells and K562-cells [40], [41]. Furthermore, cAMP can inhibit contractile machinery in endothelial cells in during different circumstances. For example, LPS induces cell contraction [42] and thrombin-induced endothelial hyperpermeability [43], [44].

In this study, we have found a β-adrenergic pathway that is distinct from the adrenergic glucose uptake pathway described in previous papers [5], [17]. By using pharmacological inhibitors we demonstrate that neither PKA, Epac, PKG, PI3K nor PKC are involved. The isoprenaline effect was not reduced by the inhibitors although two of them (4-CM and KT5823) could partly mimic the isoprenaline effect by themselves. This would either be due to that PKA and PKG are positively regulating contraction or that inhibitors exert unspecific effects leading to inhibition of contraction. The main conclusions from these experiments are that the β-adrenergic effect studied in this paper does not follow the classical β-AR signaling pathway.

β-adrenergic inhibition of contraction is a well-known phenomenon in smooth muscle. Smooth muscle myosin and non-muscle myosin have similar regulation through phosphorylation of the myosin regulatory light chain. This is distinct from cardiac and skeletal muscle myosin which is regulated by the troponin/tropomyosin complex. Thus we find it likely that β-adrenergic stimuli could affect both smooth muscle myosin and non-muscle myosin and in similar manner.

There are different proposed models for the β-adrenergic action in smooth muscles and the exact mechanism seems to vary between species [45]. In porcine and bovine airway smooth muscle, isoprenaline is shown to act both by modulation of Ca^2+^-flux via SERCA as well as by increasing activity of the myosin light chain phosphatase (MLCP) [46]. Another suggested mechanism is opening of potassium-channels (reviewed in [47]). In smooth muscle systems is shown that β-adrenergic stimulation induces membrane hyperpolarization via opening of K^+^-channels [4], [27], [28].

Recent studies have revealed that β-AR can activate various classes of potassium channels [4], [26]–[28]. Thus, we continued investigating the potential involvement of such channels in our system. Potassium channels have different numbers of transmembrane domain (TM) depending on the class but all of them have a tetrameric structure which is arranged in four subunits around a central ion conducting pore (P), forming both homotetramers and heterotetramers. There are four major classes of potassium channels: calcium-activated potassium channels, which respond to calcium or other signaling molecules; inwardly rectifying potassium channels, which conduct potassium ions more easily into the cell; tandem pore domain potassium channels, which are constitutively open or more active and contribute to the resting potential, and; voltage-gated potassium channels, which function to regulate membrane potential by repolarization of the action potential in nerve cells.

β-ARs have been shown to activate different potassium channels in different tissues. There is evidence that cAMP can directly induce the activation of potassium channels [48]. We therefore tested whether β-AR signaling via cAMP could act directly on potassium-channels and prevent cell detachment and contractility in response to calcium depletion. By using different pharmacological approaches such as inhibition of potassium channel activity with TEA or activate with pinacidil we showed that the isoprenaline effect is mainly dependent on potassium channels. In narrowing down the candidates to a class of potassium channels, we ruled out the involvement of ATP-sensitive potassium channels and calcium-activated potassium channels by showing that the inhibitors glibenclamide and charybdotoxin did not reduce the effect of isoprenaline on cell detachment and contractility. Further elucidation of the involved potassium channel through electrophysiology and molecular remains more than a trivial undertaking, as circa 80 potassium channels have been identified. Furthermore, the pharmacological profile is complicated and it is not current possible to only inhibit a specific class of potassium channel.

One possible effect on calcium depletion could be changes in the intracellular calcium levels. Here we show that in the presence of EDTA, there is a tendency towards decreased intracellular calcium in the L6 myotubes. This effect was however not changed by β-adrenergic stimulation. Thus, even if EDTA might affect intracellular calcium levels, this is not the effect that β-adrenergic signaling interferes with. Instead it is more likely to interfere with the depolarization caused by calcium removal. Depolarization can be counteracted by opening of potassium channels, and this event is, according to our present findings, promoted by cAMP that is produced downstream of the β_2_-receptor.

Sympathetic signaling is a part of the autonomic nervous system and regulates almost all organs in the body, leading to e.g. accelerating heart rate, releasing triglycerides from fat and glucose from liver, increasing blood flow and glucose uptake in skeletal muscle, dilating pupils and airways, and reducing gut movements. It is possible that regulation of non-muscle myosin is involved in several of these processes both in normal physiology and pathophysiology. For example, β-adrenergic inhibitor of contractility is a known phenomenon for smooth muscle cells, but we show here that it is likely that β-adrenergic signaling can regulate the myosin-machinery in several tissues. Myosin-dependent contraction is involved in a multitude of physiological processes such as cell shape, adhesion and tissue architecture. Enhanced myosin-dependent contraction is also associated with several diseases such as Crohn's disease, NSAID-associated enteritis, diarrheal syndromes as well as different evens during cancer such as invasion, metastasis and contraction of endothelial cells allowing intravasation of tumor cells [49], [50]. The calcium depletion model that is used in the current paper to activate myosin has previously been used to mimic the state of increased myosin-contraction that occurs during those diseases [7], [14]. Interestingly, in the current paper we show that the kinase ROCK is activated during calcium-depletion, and this kinase is shown to plays an essential part in tumor cell invasion and has been suggested as a potential therapeutic target for the prevention of cancer invasion and metastasis [51], [52], [53]. Thus, further understanding of how β-adrenergic signaling does inhibit myosin contraction could potential lead to the finding of new drug targets for several diseases.

In conclusion, we present here the novel finding that β-adrenergic signaling can greatly affect cell morphology, especially in muscle cells, and this could be of importance when studying β-adrenergic or cAMP signaling. We show that these morphological changes, induced by β-adrenergic signaling, are extremely sensitive and occur via myosin-dependent contractility. The mechanism for the inhibition of contractility by β-adrenergic agonist isoprenaline is dependent on potassium channels.

## Materials and Methods

### Cell culture

CHO-K1 and CHO-β_2_ were obtained as a gift from Roger Summers, Monash University and grown in DMEM/Hams-F12 medium supplemented with 10% FBS, 4 mM L-glutamine, 4500 mg/L D-glucose and maintained in water jacketed incubators at 37°C and 5% CO_2_.

L6 cells were purchased from American Type Culture Collection and grown in DMEM supplemented with 10% FBS, 2 mM L-Glutamine, 50 U/ml penicillin and 50 µg/ml streptomycin in a 37°C incubator with 8% CO_2_. To induce differentiation, serum levels were decreased to 2% for 7–8 days.

### Fluorescent staining

CHO-β_2_ cells were seeded out on cover slips the day before the experiment while L6 cells were grown on cover slips until reaching confluence followed by differentiation for 7 days. On the day of the experiment, cells were washed in PBS and immediately fixed (control) or kept in PBS for additionally 20 min (CHO cells) or 3 min (L6 mytoubes) in the presence or absence of 1 µM isoprenaline before fixation with 4% formaldehyde for 20 min at room temperature. Cells were permeabilized with 0.25% Triton-X100 for 10 min and FITC-conjugated phalloidin (Sigma-Aldrich; St. Louis, MO) was used to stain filamentous actin. The fluorescence was detected in a Leica DMLB epifluorescence microscope, equipped with a DC350F camera and using IM500 software (Leica Microsystems AB; Kista, Sweden).

### Adhesion assay CHO-cells

Cells were seeded out in 6-well plates and left overnight to adhere. Medium was changed to serum-free medium for 3 h. Cells were washed in PBS and kept in PBS or EDTA•trypsin as indicated.

### Adhesion assay L6 myotubes

On day 7 or 8 of differentiation, cells were incubated with inhibitors for the time indicated for each experiment, before treatment with isoprenaline (1 µM). After 30 min isoprenaline treatment, media was removed, cells washed once and then kept in trypsin•EDTA (2.5 g porcine trypsin, 0.2 g EDTA•4Na per liter HBSS), in continues presence of drugs and inhibitors, for 20 min (myotubes) or 3 min (myoblasts) at room temperature. At this timepoint, the plate was swirled gently in order to mix the detached cells, and supernatant was collected and the detached cells were counted in a single use Bürker chamber (ISL Immune Systems). Cells were pretreated with different pharmacological agents at this time at concentrations indicated in the text. All reagents were purchased form Sigma with exception of 4-cyano-3-methylisoquinoline (4-CM), which was purchased from Calbiochem, LY 294002 is from Alexis biochemicals and N6-benzoyl-cAMP and 8-pCPT-2-O-Me-cAMP from Biolog Life Science Institute. Detached cells are expressed as % of control.

### Measurement of intracellular calcium

Intracellular calcium-levels were detected by the fluorescent calcium-specfic dye Fluo-4 AM (Invitrogen). Differentiated L6 myotubes were loaded with dye according to the manufactures instructions and fluorescence was detected by in a Leica DMLB epifluorescence microscope, equipped with a DC350F camera and using IM500 software (Leica Microsystems AB; Kista, Sweden). Cells were treated with either the calcium ionophore A23187 (Sigma) or expose to EDTA in the absence or presence of isoprenaline. Flouresence intensity was quantified by the software MacBiophotonics ImageJ (McMaster Biophotonics Facility, McMaster University, Canada).

### Data Analysis

All data are expressed as mean ± s.e.mean of n. Analysis of difference was carried out with one-way ANOVA or Student t-test (Graph-pad PRISM). A value of p<0.05 was considered statistical significant.

## Supporting Information

Figure S1 EDTA-exposure induces contractility in L6 myotubes. On day seven of differentiation, culture media was removed and L6 myotubes were exposed to EDTA for 20 min. Pictures were taken every 30 second.
(MOV)

Figure S2 Isoprenaline inhibits EDTA-induced contractility in L6 cells. On day seven of differentiation, cells were pre-treated with 1 µM isoprenaline for 30 min before exposure to EDTA for 20 min, in the continuous presence of isoprenaline. Pictures were taken every 30 second.
(MOV)
